# Effects of elongation longitudinaux avec decoaption osteo-articulaire and post-facilitation stretching technique on pain and functional disability in mobile users with text neck syndrome during COVID-19 pandemic: A randomized controlled trial

**DOI:** 10.1097/MD.0000000000033073

**Published:** 2023-03-24

**Authors:** Maryam Farooq, Muhammad Salman Bashir, Abida Arif, Muhammad Kashif, Nosheen Manzoor, Farwa Abid

**Affiliations:** a Riphah College of Rehabilitation and Allied Health Sciences, Riphah International University, Islamabad, Pakistan; b School of Rehabilitation Sciences, The University of Faisalabad, Faisalabad, Pakistan; c The University of Management and Sciences, Lahore, Pakistan; d Bahria University College of Physical Therapy, BUHS Campus, Karachi, Pakistan; e Islam College of Physical Therapy, Sialkot, Pakistan.

**Keywords:** cervical pain, COVID-19, mobile phone, neck posture, stretching, text neck

## Abstract

Adolescent smartphone users have developed a variety of musculoskeletal conditions due to their use of smartphones. ELDOA (Elongation longitudinaux avec decoaption osteo-articulaire) is based on maximizing facial and spinal stretching by assuming specific posture for 1 minute and post facilitation stretching is also a type of muscle energy technique that is used for chronically shortened or tight muscles. This aimed to compare the effects of post-facilitation stretching technique and ELDOA on neck pain and functional disability in mobile users experiencing Text neck syndrome during COVID-19.

**Methods:**

This single-blinded randomized control trial with a parallel group design was conducted at the Department of Physiotherapy Safi Hospital (Faisalabad, Pakistan) from September 2021 to April 2022. Forty smartphone users between the ages of 18 and 35 who had a Neck Disability Index score of >10 due to neck pain without unilateral arm symptoms participated in the study. Of the 40 participants, twenty were randomly assigned to the ELDOA group and twenty were assigned to the post facilitation stretching group, and each group received 3 weekly sessions of treatment for 6 weeks. The Numeric Pain Rating Scale (NPRS), Neck Disability Index (NDI), and Smartphone Addiction Scale (SAS) were used to measure pain intensity, functional disability, and smartphone addiction at baseline and after 18 sessions of treatment. SPSS version 22 was used to enter and analyze the data. To find comparisons between groups an independent sample *t* test was used, and a paired sample *t* test was used to find the difference within each group.

**Results:**

Post-treatment values showed statistically significant difference between groups. ELDOA group showed greater improvement in pain (*P* < .03) with 95% CI [−1.33, −0.068] and functional disability (*P* < .05) with 95% CI [−4.44, 0.143] at 6th week. There was no statistically significant difference (*P* = .35) with 95% CI [−28.6, 10.4] between the two groups regarding smartphone addiction. The NPRS, NDI, SAS scores were significantly different within each group with *P* < .05.

**Conclusion:**

The study concluded that ELDOA method and post-facilitation stretching both were effective in treating neck pain and functional disability. However, ELDOA method was superior to post-facilitation stretching effects on neck pain and functional disability among patients with text neck syndrome.

## 1. Introduction

The current era has seen a pandemic situation caused by the Coronavirus Disease 2019 (COVID-19), which has resulted in erratic challenges around the world. To limit the spread of the virus and protect people from the harmful effects of the disease, a “lockdown” has been chosen as the first line of defense, along with other measures such as social distancing and restricting gatherings in society. This scenario has created a rise in the use of smartphones by people of all ages, creating an immense influence on education, the health sector, business sector, and social life as well. Teenagers are using cellphones more frequently, which is causing a rise in musculoskeletal issues. With the increasing size of cell phones and more screen time, postural abnormalities are becoming more prevalent.^[1]^

The improper and prolonged use of smartphones has led to a cluster of symptoms commonly known as “text neck syndrome.”^[2]^The term “text neck” was first used by Dr Dean L. Fishman to describe neck injuries caused by long periods of bending the neck forward. These injuries are caused by repeated stress on the neck.^[3]^Text Neck Syndrome is also called turtle neck posture and is caused by watching mobile phones or tablets in a flexed position for long periods of time. Text neck syndrome can cause serious damage to the cervical spine, such as inflammation of the ligaments, irritation of the nerves, and a more curved spine if it is not treated. With the growing use of technology in the modern lifestyle, this syndrome has become a new health concern.^[4]^ A smartphone user’s neck can suffer from this syndrome due to repetitive stress injury and sustained cervical flexion.^[5,6]^Studies indicate that mobile phone usage is very common among people between the ages of 18 and 44. It has been reported that 79% of the population keeps their smartphones with them almost most of the time.^[7,8]^ A common symptom of text neck syndrome is neck pain or discomfort, soreness, with other symptoms including shoulder discomfort, increased spinal curvature, and chronic headaches. Moreover, it can cause chronic low back pain in the upper back, characterized by dull pain to severe pain and muscle spasms.^[3]^

Text neck syndrome can also be considered as 21st century syndrome due to numerous reporting cases. Musculoskeletal pain especially neck pain is a public health condition in modern time.^[9,10]^ Several other factors can lead to neck pain such as neoplastic disease, inflammatory conditions, infectious conditions and congenital problems. Growing evidence reports that persistent neck pain in young age leads to chronic pain in adults.^[11–14]^ This condition is a growing concern in young population as they use smartphones or electronic gadgets for simple tasks.^[15]^

Warm-up exercises, stretches, rest, ice/heat, massage, posture correction, and lifestyle changes may help acute text neck symptoms. Chronic cases may benefit from physical therapy, medication, injections, manipulation, and acupuncture.^[4]^Outpatient physiotherapy provided by physical therapists during the COVID-19 pandemic was an important part of physical rehabilitation for patients with different Musculoskeletal, neurological, and respiratory conditions.^[16,17]^ Physiotherapy includes manual techniques used by skilled clinician to diagnose and treat soft tissues and joint structures. As part of this approach, increasing range of motion is achieved by enhancing the repair and extensibility of the surrounding tissues, facilitating movement, and decreasing disability levels by improving musculoskeletal function pertaining to daily activities.^[18]^ Post facilitation stretching is also type of muscle energy technique that is used for chronically shortened or tight muscles. It involves relaxation of muscle and lengthening of fascia.^[19]^In this application, the patient was instructed to contract at or near patient’s maximum voluntary capacity for 5 to 10 seconds and then relax as quickly as possible. The researcher would aggressively stretch the muscle at barrier.^[19]^

In text neck syndrome pain is caused by mostly by stiff trapezius and levator scapulae.^[4]^ Myofascial ELDOA (Elongation longitudinaux avec decoaption osteo-articulaire) technique was first described by Guy Voyer in 1979, which is also called LOADS (longitudinal osteoarticular decoaptation stretching).^[20]^ It is based on maximizing fascial and spinal stretching by assuming specific posture for 1 min. It targets spinal strengthening and decompression.^[21]^ ELDOA has originated from a variety of treatment approaches and creates local and general effects. It allows decompression of zygopopheasal joints, more absorption of fluids in disc, increase circulation, improve tone and end range. It also allows correction of impaired posture, improving respiration and proprioceptive facilitation of concerned segment. It has also secondary benefits on organ system.^[19,21]^A number of conditions have been treated with ELDOA, including prolapsed intervertebral discs, piriformis syndrome, chronic low back pain, cervical radiculopathy, active trigger points, and forward head posture.^[21–25]^

This syndrome has become a new health concern worldwide and affects the huge population. Adolescent smartphone users increase day by day and have a lot of musculoskeletal conditions. The size of cell phones become bigger day by day and demands more screen time. Prolonged duration of flexed posture while looking down on a screen leads to pain intensity and disability in cervical spine. Few studies have been conducted on the physical therapy management of text neck syndrome than those on prevalence and awareness. Hence, this study aimed to examine the effects of post-facilitation stretching versus ELDOA treatment on mobile users experiencing text neck syndrome during the COVID-19 pandemic.

## 2. Methods

This single-blinded randomized clinical trial was conducted at the Physical Therapy department of Safi Hospital, Faisalabad and was registered in the WHO Registry clinicaltrials.gov with number. NCT05048992 and ethical approval (REC/RCR & AHS/21/0116) was granted by Research and ethics committee of Riphah International University and this trial followed the Declaration of Helsinki.

### 2.1. Randomization

**Figure 1.**
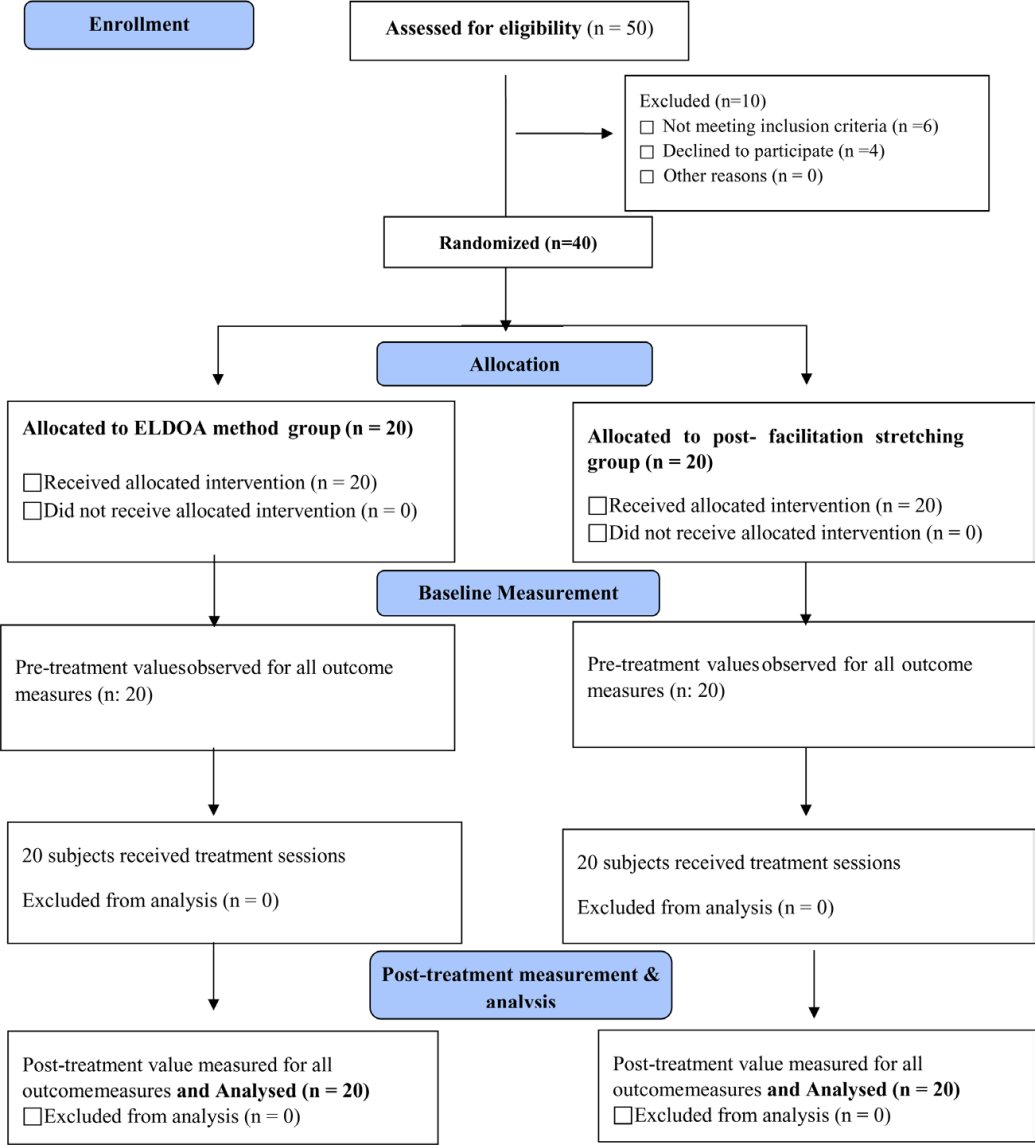
CONSORT study flow diagram.

The study recruited 40 patients (18 women, 22 men) with Text neck syndrome through consecutive sampling, and in this single-blind study, one of the groups received post-facilitation stretching, and the other received ELDOA. The study flow diagram is shown in Figure 1.

Forty patients with acute neck pain without unilateral upper extremity symptoms, 18 to 35 years of age, using smartphone since past 1 year and neck disability index (NDI) score > 10 were included in the study. Participants having any congenital cervical abnormalities, history of whiplash injury within the last 6 weeks, history of spinal neoplastic disease, spinal infection, cervical spine fracture, or previous surgical neck procedure and cervical radiculopathy were excluded from the study. Taking into account the above-mentioned inclusion and exclusion criteria, subjects were recruited in this study through consecutive sampling and then participants were randomly assigned to both groups by the researcher. After taking informed consent, subjects assigned to Group A and group B by lottery method. All participants had equal choice of group allocation and asked to draw one chit from a box. Participants with an odd number were assigned to group A and participants with even number assigned to group B.

### 2.2. Sample size

Sample size of 36 was calculated with effect size 0.99 by using G power analysis software, version (3.1.9.2)^[26]^ with 0.80 power of study, 5% margin of error and 95% confidence interval with 10% attrition rate.^[21]^

### 2.3. Treatment

Treatment was given to both groups for 6 weeks with 3 sessions per weeks on alternate days of the week with 45 minutes duration of each session and treatment session was conducted during the daytime. A pre-assessment was conducted before treatment commenced, and a post-treatment assessment was conducted at the last session, using the Numeric Pain Rating Scale (NPRS), NDI and Smartphone Addiction Scale (SAS). All patients in both groups were advised to take frequent breaks during Smartphone use and avoid static posture as a home plan.^[4]^

#### 2.3.1. Procedure.

Before each session, both groups received a 10-minute hot pack session.

#### 2.3.2. ELDOA group.

group A was treated with ELDOA which is myofascial stretching technique of the cervical spine. Patients were asked to adopt desired position to target intervertebral segments. Breathing is also important factor in ELDOA stretch. Patients were instructed to not to hold or strain breathing through promoting diaphragmatic breathing.

#### 2.3.3. Level CO-C2.

The patient was inside lying with bottom limb place below the head along with neutral cervical spine. Bottom leg was bent to stabilize the trunk. Progression of this posture was made through lengthening of neck and rest of the spine. Patients were asked to maintain this position for 1 minute. After that Patients were asked to extend top arm over head with palm placed on the ground. Foot of top leg placed anterior to the knee by keeping hip in vertical position. Patients were asked to maintain this position. In the final step, Patients were asked to protrude their tongue and move their eyes towards head.

#### 2.3.4. Level C4-C5.

The starting position of patients was supine lying with both arms placed on the sides. Patients were asked to move to both knees towards chest until tension reaches the sacrum region. Then Patients were asked to raise their head from floor to produce further tension. Progression of this position was made through extension of both arms along with external rotation at shoulder. Patients were asked to maintain this position for 1 minute.

#### 2.3.5. Level C5-C6.

This is the progression of level C4-C5. For targeting C5-C6, patients were asked to rotate both arms toward ceiling by keeping in parallel direction. Tension was maintained for 1 minute.

#### 2.3.6. Level C6-C7.

This is the progression of level C5-C6. Patients were asked to abduct both arms in coronal plane up to 45 degrees. Tension was maintained for 1 minute.^[19]^ Every posture was maintained for 1 minute with 15 seconds rest and each session consists of 5 repetitions.^[19]^

#### 2.3.7. Post-facilitation stretching technique group.

Post facilitation technique was used to treat tight trapezius and levator scapulae. At first patient was instructed to contract at or near patient’s maximum voluntary capacity for 5 to 10 second and then relax as quickly as possible. The researcher was aggressively stretch the muscle at barrier.^[7]^

#### 2.3.8. Levator scapulae.

Each patient was in supine position. Patients were asked to place their affected hand under the thigh in order to produce shoulder depression. Therapist was standing towards the head of patient and placed hand on non-affected side. Therapist attempted to flex, contra-laterally side flexion and rotation until tissue resistance was felt. After that therapist placed the muscle in mid-range and lengthened aggressively beyond tissue resistance with 15 second hold. After that new barrier was achieved and same procedure repeated.

#### 2.3.9. Upper trapezius.

Patients were asked to place their affected hand under the thigh in order to produce shoulder depression. Therapist was standing towards the head of patient and placed hand on non-affected side. Therapist attempted to flex, contralaterally side flexion and ipsilateral rotation until tissue resistance was felt. After that therapist placed the muscle in mid-range and lengthened aggressively beyond tissue resistance with 15 second hold. After that new barrier was achieved and same procedure repeated.^[19]^Each session consists of 5 repetitions. Treatment was given to both groups for 6 weeks with 3 sessions per weeks. All patients were advised to take frequent breaks during smartphone use and avoid static posture as a home plan.

#### 2.3.10. Outcome measures.

The primary outcomes were:

• The intensity of pain was assessed using the NPRS, which is valid, reliable, and universally accepted scale.^[27]^ It consists of 11 points. In which 0 means no pain and 10 means severe pain.^[28]^
• This study measured functional neck disability using NDI, which is widely used and well validated.^[29]^ It is scale of ten sections with each section of 5 marks. Interpretation of this scale is as 0 score indicates no activity limitation and 5 score indicates complete activity limitation. Score ranges from 0 to 50 in which 0 indicates no disability and 50 means maximum disability. A higher score indicated greater level of activity limitation.^[29]^

The secondary outcome was:

• An assessment of smartphone addiction was performed using the SAS, which has been proven to be a reliable and valid instrument. Total score of SAS ranges between 33 to 198 and higher score indicates more disability.^[30]^

The study was single blinded as only the assessor was blinded during treatment, and patients and the clinical therapist were not blinded due to the nature of the treatment protocol.

### 2.4. Statistical analysis and interpretation

The data analysis was done by using SPSS version 22 for Windows software. For descriptive statistics frequency tables and bar charts were used. Parametric test was used to compare two population at different various intervals. To find between group comparison independent sample *t* test was used independent sample *t* test was applied to measure difference between two groups. To find difference within each group Paired sample *t* test was used.

## 3. Results

The mean age of participants in ELDOA group was 21.30 ± 1.97 and in post–facilitation stretching technique group 21.05 ± 1.76 with *P* value .67. The body mass index of participants in ELDOA group was 21.29 ± 4.14 and in post–facilitation stretching technique group 22.05 ± 4.24 with *P* value .56. The Pre-NPRS score in ELDOA group was 4.80 ± 1.36 in post-facilitation stretching group was 4.4 ± 1.18 with *P* value.32. The Pre-NDI score in ELDOA group was 16.20 ± 5.18in post–facilitation stretching group was14.65 ± 4.71with *P* value .32. The Pre-SAS score in ELDOA group was 111.7 ± 35.7 score in post–facilitation stretching group was 118.4 ± 20.25 with *P* value .47. The independent *t* test and levene’s test for equality of variances was used to evaluate homogeneity of among two groups (Table 1).

**Table 1:** General characteristics of participants.

Study group	ELDOA method (n = 20)	Post–facilitation stretching technique (n = 20)	*P* value
	(Mean ± SD)	(Mean ± SD)	
Age of participants	21.30 ± 1.97	21.05 ± 1.76	.67
Height in m	1.63 ± 3.70	1.67 ± 2.44	.42
Weight in kg	56.23 ± 20.56	59.78 ± 23.0	.26
Body mass index of participants	21.29 ± 4.14	22.05 ± 4.24	.56
Pre-NPRS	4.80 ± 1.36	4.4 ± 1.18	.32
Pre-NDI	16.20 ± 5.18	14.65 ± 4.71	.32
Pre-SAS	111.7 ± 35.7	118.4 ± 20.25	.47

ELDOA = elongation longitudinaux avec decoaption osteo articulaire, NDI = neck disability index, NPRS = Numeric Pain Rating Scale, SAS = Smartphone Addiction Scale.

Out of 40 participants, 20 subjects were randomly allocated to ELDOA group and 20 were assigned to post facilitation stretching group. There is no dropout reported in each group. Therefore, data of all participants was included in the analysis. Normality of data was assessed by Shapiro–wilks test. Data was normally distributed because *P* value was > .05. Parametric tests were used to compare groups on baseline and post-treatment values. Between groups comparison was performed by independent t test on different outcome measures. Pre values of both groups were comparable on NPRS, Neck disability index (NDI) and SAS (*P* > .05).The finding of the current study revealed that there was a significant difference between the groups on NPRS and NDI pre- and post-treatment for pain and functional disability with a *P* value of (*P* < .03) with 95% CI [−1.33, −0.068] and (*P* < .05) with 95% CI [−4.44, 0.143], respectively, and ELDOA group showed greater improvement in functional disability (*P* < .05). However, between group comparison of SAS score reported no significant difference (*P* = .35) after treatment with 95% CI [−28.6, 10.4] (Table 2). Within group comparison of NPRS, NDI, and SAS scores showed significant difference *P* < .05 and 95% CI [LL, UL] values are mentioned in within group comparison table (Table 3).

**Table 2:** Between group comparison of NPRS, NDI and SAS score.

Outcomes measures	Study groups		*t* test		
	ELDOA	PSF	*P* value	95% confidence interval of the difference	
	Mean ± SD	Mean ± SD		Lower	Upper
Baseline NPRS	4.80 ± 1.36	4.40 ± 1.18	.32	−0.42	1.217
6th week NPRS	1.70 ± 1.03	2.40 ± 0.94	.03	−1.33	−0.068
Baseline NDI	16.2 ± 5.18	14.6 ± 4.71	.32	−1.62	4.723
6th week NDI	5.70 ± 3.14	8.80 ± 3.44	.005	−4.44	0.143
Baseline SAS	111.70 ± 35.7	118.4 ± 20.2	.47	−25.3	11.9
6th week SAS	80.65 ± 34.5	89.7 ± 25.67	.35	−28.6	10.4

ELDOA = elongation longitudinaux avec decoaption osteo articulaire, NDI = neck disability index, NPRS = Numeric Pain Rating Scale, PSF = post-facilitation stretching, SAS = Smartphone Addiction Scale.

**Table 3:** Within-group comparison of NPRS, NDI, and SAS.

Outcome measures	Groups	Baseline	6th week	95% confidence interval of the difference		Paired *t* test		
		Mean ± SD	Mean ± SD	Lower	Upper	*t*	df	*P* value
NPRS	ELDOA	4.80 ± 1.36	3.15 ± 0.67	2.83605	3.46395	21	19	<.001
	Post-facilitation stretching	4.40 ± 1.18	2.00 ± 0.72	1.66047	2.33953	12.3	19	<.001
NDI	ELDOA	16.2 ± 5.18	10.50 ± 2.94	9.12081	11.8792	15.9	19	<.001
	Post-facilitation stretching	14.6 ± 4.71	5.85 ± 2.03	4.8984	6.8016	12.9	19	<.001
SAS	ELDOA	111.70 ± 35.7	31.05 ± 11.29	25.7649	36.3351	12.3	19	<.001
	Post-facilitation stretching	118.4 ± 20.2	28.65 ± 11.28	23.3675	33.9325	11.4	19	<.001

ELDOA = elongation longitudinaux avec decoaption osteo articulaire, NDI = neck disability index, NPRS = Numeric Pain Rating Scale, SAS = Smartphone Addiction Scale.

## 4. Discussion

Excessive use of smartphone use may affect behavioral, physical, and musculoskeletal health, including head and neck issues.^[31]^ The aim of the study was to determine the effects of ELDOA and post facilitation stretching technique on neck pain and disability among patients with text neck syndrome. Neck pain is a common problem now days and associated poor posture while using smartphones or laptops.^[32,33]^This syndrome spread globally during the COVID-19 pandemic as all work shifted to laptops or smartphone.

In this study, ELDOA method which is myofascial stretching technique in comparison with the post facilitation stretching technique was administrated to study groups. In 2018, Stępnik conducted a study on tissue targeted manual techniques which emphasized on fascia of neck region to reduce neck pain and disability. The study was performed on 31 participants with age 25 to 53 years allocated to experimental and control group. Five manual techniques were assigned to experimental group and laser therapy to the control group. Assessment was performed by Neck disability index. The study concluded that there is statistically significant reduction in neck pain and disability up to 8.5 points. Patients reported improvement in ADL’s.^[34]^ As in this study, ELDOA method reported same outcomes because ELDOA is a longitudinal Osteoarthritis decoaptation technique which targets fascia by maximizing tension. The difference from this study was age group 25 to 53 years. Javaid et al conducted a study on effectiveness of ELDOA method for trigger points in upper trapezius and levator scapulae. They concluded ELDOA was helpful in reducing pain, discomfort, disability and improving neck related disabilities.^[24]^

ELDOA method is basically postural exercises that could be taught to the patients. These exercises separate the vertebral spaces by generating facial tension at targeted vertebral segment. Separation causes increase in blood flow, disc hydration. Muscles strengthening, improvement in proprioception also achieved through these postural exercises. Retamal et al^[35]^ conducted a trial to determine the effectiveness of both instrumental and manual soft tissue techniques in sub-occipital region of neck among patients with chronic mechanical neck pain. They concluded that both instrumental and manual soft tissue approaches showed equal improvement in neck pain and disability. General improvement was seen in all parameters (*P* < .05) and between group comparison showed no significant results (*P* > .05). In our study ELDOA which was type of manual soft tissue approach was found more superior than post-facilitation stretching. Another study conducted on the effects of modified ELDOA and ELDOA on cervical radiculopathy reported that both interventions were effective in improving posture and disabilities; however, no significant difference was found.^[23]^

Brück et al^[36]^ conducted a clinical trial in 2021 to compare the effects of fascial therapy and manual therapy among patients of chronic neck pain. In the past literature fascia targeted therapy grabs the attention of physiotherapists as it has beneficial effects on chronic neck pain. This study was conducted on three groups, Fascial therapy group, Manual therapy group and no treatment group. Subjects were allocated to three groups and assessed by using, visual analogue scale and Neck pain and disability scale. Results reported large effect size of fascial therapy and manual therapy in chronic neck pain. As in this study, both interventions reported improvement in neck pain and disability. The difference between this study and our study is that we have two groups whereas this study conducted on three groups.

In 2021 Raja et al^[37]^ performed a RCT to determine the effectiveness of yoga poses and deep cervical fascial therapy on mechanical neck pain. Patients were recruited and assessed on NPRS, patient specific functional scale and neurodynamic test. They concluded in favor of deep cervical fascial approach and yoga as a promising treatment protocol for mechanical neck pain. Fascia is an important factor in treating musculoskeletal problem and in this study, results supported that ELDOA method (myofascial stretches) are more effective as compared to post- facilitation stretching. Due to the pandemic, patients were reluctant to participate so it was more difficult to instruct those regarding different ELDOA postures and giving verbal and tactile cues to hold postures. More research may be necessary to determine the longevity effects of interventions with follow-up sessions and objective measures, as well as on other symptoms of text neck syndrome. More studies with a larger sample size and adding patient with chronic condition should be conducted in the future by the researchers. It is recommended that clinicians use the ELDOA method in routine practice since it provided better results than post-facilitation stretching in reducing neck pain and functional disability among patients with Text Neck Syndrome.

## 5. Conclusion

The study concluded that the ELDOA method and post-facilitation stretching were both effective in treating neck pain and functional disability. However, the ELDOA method provided superior results to post-facilitation stretching for neck pain and functional disability among patients with Text Neck Syndrome. Furthermore, no significant difference was found between groups in smart phone addiction outcomes.

## Author contributions

**Conceptualization:** Maryam Farooq, Muhammad Salman Bashir.

**Data curation:** Maryam Farooq, Muhammad Kashif.

**Formal analysis:** Maryam Farooq.

**Funding acquisition:** Maryam Farooq, Abida Arif, Nosheen Manzoor, Farwa Abid.

**Investigation:** Maryam Farooq, Abida Arif, Nosheen Manzoor.

**Methodology:** Maryam Farooq, Abida Arif, Muhammad Kashif, Farwa Abid.

**Project administration:** Muhammad Salman Bashir.

**Resources:** Maryam Farooq, Abida Arif, Nosheen Manzoor, Farwa Abid.

**Software:** Abida Arif, Nosheen Manzoor, Farwa Abid.

**Supervision:** Muhammad Salman Bashir.

**Validation:** Muhammad Salman Bashir, Muhammad Kashif.

**Visualization:** Maryam Farooq, Muhammad Salman Bashir, Muhammad Kashif.

**Writing – original draft:** Maryam Farooq, Muhammad Salman Bashir, Muhammad Kashif.

**Writing – review & editing:** Maryam Farooq, Muhammad Salman Bashir, Muhammad Kashif.
